# Simultaneous Tumor and Stroma Targeting by Oncolytic Viruses

**DOI:** 10.3390/biomedicines8110474

**Published:** 2020-11-05

**Authors:** Anne Everts, Melissa Bergeman, Grant McFadden, Vera Kemp

**Affiliations:** 1 Research Program Infection and Immunity, Utrecht University, 3584 CS Utrecht, The Netherlands; 2 Center for Immunotherapy, Vaccines and Virotherapy (CIVV), The Biodesign Institute, Arizona State University, Tempe, AZ 85287, USA; mhbergem@asu.edu (M.B.); grantmcf@asu.edu (G.M.); 3 Department of Cell and Chemical Biology, Leiden University Medical Center, 2333 ZC Leiden, The Netherlands; V.Kemp@lumc.nl

**Keywords:** tumor stroma, tumor microenvironment, oncolytic viruses, virotherapy

## Abstract

Current cancer therapeutics often insufficiently eradicate malignant cells due to the surrounding dense tumor stroma. This multi-componential tissue consists of mainly cancer-associated fibroblasts, the (compact) extracellular matrix, tumor vasculature, and tumor-associated macrophages, which all exert crucial roles in maintaining a pro-tumoral niche. Their continuous complex interactions with tumor cells promote tumor progression and metastasis, emphasizing the challenges in tumor therapy development. Over the last decade, advances in oncolytic virotherapy have shown that oncolytic viruses (OVs) are a promising multi-faceted therapeutic platform for simultaneous tumor and stroma targeting. In addition to promoting tumor cell oncolysis and systemic anti-tumor immunity, accumulating data suggest that OVs can also directly target stromal components, facilitating OV replication and spread, as well as promoting anti-tumor activity. This review provides a comprehensive overview of the interactions between native and genetically modified OVs and the different targetable tumor stromal components, and outlines strategies to improve stroma targeting by OVs.

## 1. Introduction

Despite decades of research into cancer, and the development of a tremendous amount of treatment options, cancer remains a leading cause of death, with approximately 9.6 million deaths worldwide in 2018 [1]. Traditionally, a tumor has been interpreted as a single bulk of rapidly multiplying and differentiating malignant cells. To address this clump of tumor cells, patients are conventionally treated with either non-specific chemotherapy, radiotherapy, surgery, or a combination of these treatments. In addition, tumor-targeted therapies such as angiogenesis inhibitors and immunotherapies have been developed [2,3,4]. However, a variety of studies have revealed the off-target effects of these treatments on healthy non-malignant cells, resulting in severe adverse effects in patients [5,6]. Moreover, the complexity, heterogeneity and plasticity of tumor cells and the microenvironment can contribute to drug resistance and relapse [7].

A major determinant of therapeutic resistance is the dense stromal tissue surrounding and imbedding the malignant cancer cells. Solid tumors are composed of tumor cells and up to 90% of non-epithelial cells that collectively form the tumor stroma [8]. The tumor stroma is critically involved in tumorigenesis, tumor progression, dissemination and therapy resistance, also acting as a barrier for anti-tumor drugs and infiltrating immune cells [9]. In addition, this barrier has immunosuppressive capacities, possibly reducing the sensitivity of tumors to immunotherapeutics [10]. The significant impact of the stromal mass on tumor progression has opened an increasingly important cancer research area, drastically changing the rationale for anti-tumor treatment. However, the current therapeutic strategies largely neglect the complex stromal barrier surrounding tumor cells, highlighting the need for novel therapeutic options combining tumor and stroma targeting.

Oncolytic virotherapy (OV therapy) is a novel and promising therapeutic approach that has recently gained interest for its multi-faceted mechanism of action that induces immunogenic cell death and thereby strong anti-cancer immune responses. Oncolytic viruses (OVs) selectively target, replicate in and kill tumor cells while sparing normal tissues. In fact, OVs benefit from the tumor’s altered microenvironment, which displays certain tumor-specific receptors and modified cellular pathways [10,11]. OVs are often targeted and armed by means of genetic modifications that facilitate replication within cancer cells and express specific transgenes that result in enhanced tumor specificity, infectivity, replication, oncolysis, anti-tumor immunity, anti-angiogenetic effects, and/or stroma targeting. As a result, OVs are capable of modulating the immune infiltrate within the tumor stroma [10,11,12,13]. The application of such engineered OVs is a fast-growing field of research, in which multiple clinical monotherapies have been developed [12,14]. Therefore, OVs represent a promising therapeutic platform for simultaneous tumor and stroma targeting [12,13].

In this literature review, we will discuss the OVs that specifically target tumor stroma components and outline possibilities to improve this targeting, which is ultimately expected to result in enhanced anti-cancer activity.

## 2. Oncolytic Viral Therapy

OVs are a collection of ‘cancer-killing’ viruses, known for their multi-faceted attack on tumors and their components. Through direct cancer cell lysis, they can boost a more effective anti-tumor immune response [15,16]. OVs accomplish this by inducing immunogenic cell death, leading to the release of danger-associated molecular patterns (DAMPs), recruitment and activation of leukocytes, and modulation of the tumor microenvironment (TME) [17,18]. The overall inherent tumor cell binding and entry capacity of OVs are determined by the (upregulated) expression of tumor cell surface receptors that interact with virion components that mediate virus binding and entry [14]. In addition, OVs can hijack specific pathways of the tumor cells, whose deregulation promotes tumor progression and immune evasion, but also facilitates virus spread, replication, and oncolysis [14]. Examples of these are genetic defects in the protein kinase R (PKR), type I interferon (IFN), and tumor necrosis factor (TNF) pathways [14,19,20,21,22].

A large variety of oncolytic viral families have been investigated in various preclinical and clinical cancer studies [23]. These cover both single-stranded and double-stranded RNA and DNA viruses, including measles virus, vesicular stomatitis virus (VSV), reovirus, type 1 herpes simplex s (HSV-1), Newcastle disease virus (NDV), adenovirus, and poxviruses (vaccinia and myxoma viruses) [24,25,26,27,28,29,30,31,32,33]. As reviewed by Yang and colleagues, multiple viral and tumoral factors can impact OV therapy [24], but there are no general rules to predict the oncolytic efficiency of any OV with any target cancer, in part because the tumor microenvironment can vary widely, even within a single cancer patient.

### 2.1. Tumor Stromal Components

**Figure 1:**
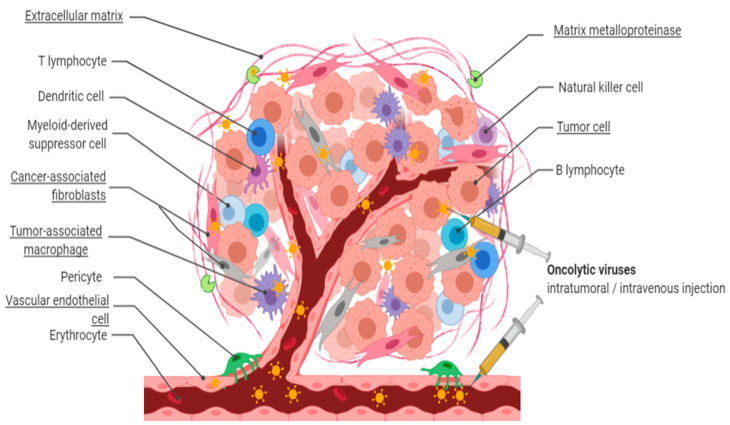
Simultaneous tumor and stroma targeting upon in situ oncolytic virus administration. In situ administration of natural, bioselected, or genetically engineered oncolytic viruses (OVs) results in targeting of tumor cells as well as stroma components, including heterogeneous cancer-associated fibroblasts, extracellular matrix, tumor vasculature, and tumor-associated macrophages. As a result, OV penetration and replication are increased, leading to enhanced tumor killing and anti-tumoral immune activity.

Solid tumors secrete cytokines to suppress the anti-tumor functions of immune cells and recruit stromal cells, forming a desmoplastic physical stromal barrier [24]. This multi-componential stroma guides the alteration and behavior of tumor cells through continuous interactions with the tumor cells as well as immune cells [10]. Consequently, the TME is commonly modified metabolically, chemically, physically, and immunologically into a niche that promotes tumor progression and metastasis [34]. Therapy resistance and tumor recurrence could be overcome by controlling the interaction between the tumor and stromal components using OV therapeutics. The major tumor stromal components which are associated with these modifications, and which are demonstrated to be promising (direct or indirect) targets for OV therapies, are outlined below. These stromal components include cancer-associated fibroblasts (CAFs), (dense) extracellular matrix (ECM), tumor vasculature, and tumor-associated macrophages (TAMs), visualized in Figure 1 [35,36]. The subject of regulatory T cells (Tregs) is beyond the scope of this review.

### 2.2. Cancer-Associated Fibroblasts (CAFs)

Fibroblasts are the major multi-functional component of tumor stroma. It has been shown that local fibroblasts can develop into CAFs, although additional CAF progenitors have been identified as well, such as mesenchymal stem cells, adipocytes, epithelial and endothelial cells [37]. CAFs steer the behavior of stromal and tumor cells, and alter the physical and biochemical stromal structure via direct cell–cell interactions and secretion of cytokines, sugars, proteins and proteases [38]. Additionally, CAFs play an important role in tumor angiogenesis, proliferation and invasion, as they produce autocrine and paracrine regulatory factors with tumor-promoting functions, e.g., vascular-endothelial growth factor (VEGF) and stromal-derived factor-1 (SDF1) stimulate the recruitment of endothelial cells [37,38]. Moreover, CAFs produce matrix metalloproteases 1 and 2 (MMP1 and MMP2) and FGF2, shaping the ECM structure and promoting tumor cell survival, respectively [39,40,41]. Specifically, CAFs were shown to be the major producer of MMP2 in murine lung tumors [41]. Furthermore, CAFs establish an immunosuppressive stroma by expressing and secreting multiple factors that hamper the survival and mobilization of anti-tumor immune cells. A large variety of underlying mechanisms have been identified, such as recruiting myeloid-derived suppressor cells (MDSCs) and regulatory T cells, as described in a recent review of Monteran and Erez [40,42]. The multitude of these functions and roles by CAFs can be accounted for by the fact that they are a heterogenous population within the tumor stroma. Additional research has begun to determine the different ways that the types of CAF subsets work to support the tumor and each other.

In recent years, interest has increased in the heterogeneity of the CAF cell population. The numerous sources of origin and the tumor-distinctive activation processes result in a large variety of phenotypic markers expressed by the CAFs [37,38,43]. Some of the notable examples of these markers are α-smooth muscle actin (αSMA) and fibroblast activation protein (FAP) [37]. Moreover, the urokinase-type plasminogen activator receptor (uPAR) is abundantly expressed by CAFs [44]. Importantly, these markers have defined biological functions and are not necessarily CAF specific, since they are often also expressed by other cells present in the tumor stroma [37]. For example, uPAR can also be expressed on vascular endothelial cells (VECs), TAMS, and tumor-infiltrating lymphocytes, via which it plays a role in tumor–stroma interactions, tumor progression and metastasis [44]. Moreover, some CAF subsets clearly exert tumor-promoting functions, whereas others seem to be tumor controlling [45]. This possibly explains the adverse and contradictory results of depleting CAFs based on surface markers such as αSMA or FAP [37]. The different stromal markers in specific cancer models, their biological functions, and co-expression in other cells have been extensively reviewed by Chen and Song [37].

#### Cancer-Associated Fibroblast Subsets (CAF Subsets)

Five different CAF subsets have been distinguished based on the origin of the precursor fibroblasts, activation stages, surface markers and secretory phenotype. An overview of the characteristics and functions of these CAF subsets is provided in Table 1. Importantly, the ‘myofibroblastic CAFs’ (myCAFs) have been shown to restrain tumor growth, whereas the other subsets all seem to exert pro-tumoral functions (Table 1) [45,46,47,48,49,50]. The tumor-promoting ‘inflammatory CAFs’ (iCAFs) have been shown to secrete tumorigenic cytokines, and hyaluronan synthase, an enzyme which promotes metastasis and therapeutic resistance [45,47,49]. The ‘antigen-presenting CAFs’ (apCAFs) are believed to enhance regulatory T cell formation and thereby dampen immune activation [45]. By doing so, these CAFs increase tumor survival by reducing the immune response to and targeting of the tumor. The ‘circulating CAFs’ (cCAFs) play an important role in metastasis and the formation of a suitable niche for future tumor cell colonization [48]. The CD10+GPR77+ CAFs are related to poor patient survival, enhanced chemoresistance and cancer stemness [50]. Altogether, the variety of CAF functions highlights the importance of targeting specific CAF subsets instead of all CAFs that might be present in the stroma. A better functional characterization of the subsets could accelerate the future design of optimized CAF-directed OV cancer therapeutics.

### 2.3. The Dense Extracellular Matrix (ECM)

The ECM comprises up to 60% of the solid tumor mass and is generated by CAFs and, to a lesser extent, tumor cells [51]. The dense ECM is disordered and stiffened by multiple factors such as the collagenous matrix, proteoglycans, and hyaluronan. The significant accumulation of the collagenous matrix alone is associated with systemic therapy resistance and poor prognosis. The excessive accumulation of these factors together results in an impermeable and rigid ECM, forming a shield surrounding the tumor cells. In addition to impeding drug permeability, it interferes with oxygenation, metabolites and nutrient supply. A reduced nutritional supply activates the pathological signaling cascades related to hypoxia and metabolic stress, resulting in activation of drug efflux pumps, reduced apoptosis, and senescence, as described by Ergün and colleagues [51]. The ECM also harbors several proteases, such as MMPs secreted by CAFs, that are associated with poor prognosis due to their capability to remodel the ECM components to enhance for example angiogenesis and tumor cell migration [51]. Altogether, the multi-componential dense ECM plays a significant role in current tumor therapy resistance and emphasizes the importance of targeting the ECM components for optimal anti-tumor treatment effects.

### 2.4. The Tumor Vasculature

Vascular endothelial cells (VECs) can be transformed into more rapidly proliferating and migrating tumor endothelial cells (TECs) upon specific targeting and stimulation of signaling pathways by tumor cells and surrounding activated stromal cells [52]. Together, these cells represent the tumor vasculature, which provides nutrients for tumor growth and facilitates angiogenesis [53]. The continuous tumor–endothelial cell interactions induce and regulate secretion of adhesion molecules, chemokines, and excessive levels of VEGF by tumor cells and CAFs. This results in enlarged density regions, hypoxia-induced angiogenesis, and vessel hyperpermeability, leading to an abnormal vessel structure, metastasis and eventually therapy resistance [37,52]. These described co-interactions between the tumor vasculature and tumor cells highlight the importance of targeting both components to enhance anti-tumor therapeutic effects.

### 2.5. Tumor-Associated Macrophages (TAMs)

Tumors steer both the innate and adaptive immune cell composition of the TME. In solid tumors, half of the tumor mass is represented by types of macrophages known as TAMs, which polarize into the pro-tumoral and anti-inflammatory M2 phenotype. The described recruitment and polarization are promoted by the tumor and immune cell secreted cytokines, growth factors and metabolites such as VEGF, colony stimulating factor-1 (CSF-1) and chemokine CCL2 [54]. TAMs can be distinguished from the M1 phenotype based on their specific markers, including CD163, CD204, and CD200R, and their upregulated genes, such as arginase-1 and macrophage mannose receptor [55]. Moreover, TAMs secrete pro-inflammatory cytokines which affect multiple aspects in tumor progression such as suppressing anti-tumor immune cells, enhancing tumor cell proliferation, stimulating angiogenesis, and promoting metastasis [56]. More specifically, TAMs promote stiffening and fibrosis of the ECM by secreting TGF-𝛽, which in turn is shown to stimulate production and cross-linking of collagen [9]. Altogether, large TAM infiltration results in complex pro-tumoral interplay with the tumor cells and other stromal components, which is related to poor prognosis [57]. To date, the body of OV literature lacks direct TAM targeting by OVs, as it only describes the indirect effects of OVs on TAMs. However, the above discussed pro-tumoral interplay emphasizes the need to, and possible promising effect of, future OVs targeting TAMs specifically.

## 3. Natural Stroma Targeting by Oncolytic Viruses

Several studies have demonstrated the inherent capability of OVs to target the tumor stroma. Below, an overview is provided on the different stromal components for which natural targeting by OVs has been described.

### 3.1. Natural Targeting of Cancer-Associated Fibroblasts (CAFs)

CAFs distinguish themselves from normal cells through their cellular origins, phenotypic plasticity, and diverse functions, as previously discussed. To date, natural CAF tropism has been identified for only one OV: VSV. VSV has been shown to successfully infect CAFs in patient-derived xenograft models. VSV replication in pancreatic tumor cells and stroma is enhanced by the cross-talk between tumor cells and CAFs [58]. This cross-talk occurs when the tumor cells secrete TGF-β1, which promotes VSV infection in CAFs, and CAFs secrete FGF2, which reduces innate anti-viral retinoic acid-inducible gene I (RIG-I) expression in pancreatic tumor cells. The reduced expression of RIG-I makes these cells more permissive to viral infection. As a result of this cross-talk, a niche consisting of VSV-sensitive tumor cells and CAFs is maintained [58]. However, the targeting of a specific CAF subset has not yet been described. Therefore, it remains to be determined whether VSV and other OVs have a preference for specific subtypes of CAFs. As CAF subsets exert different defined functions, this could greatly influence the therapeutic relevance of the OV-mediated targeting.

### 3.2. Natural Targeting of the Tumor Vasculature

Four OVs with natural VEC specificity or binding abilities have been described, including reovirus, VSV, HSV, and vaccinia virus (VV). Wild-type reovirus has been found to effectively infect and lyse vascular endothelial cells (VECs) upon artificial elevation of VEGF levels. Subsequently, reoviral delivery and tumor lysis was enhanced in vitro and in murine syngeneic melanoma tumor models [59]. Similar effects were seen with VSV, which showed improved overall survival of mice. Long-term cure was observed upon reovirus treatment but not with VSV treatment. An important concern is that reoviral replication also occurred in non-tumor tissues in mice, although no overt toxicity was detected [59]. The effects on these non-tumor cells specifically remain to be further elucidated.

The 3D imaging of a murine colon cancer model has revealed direct VSV infection of VECs in tumor vasculature, but not normal vasculature. VSV infection induced coagulation in the neovasculature, reducing tumor perfusion and proliferation of malignant cells within the tumor core [60]. The mechanism of VEC infectivity by VSVs remains to be studied, in which the role of the above discussed VEGF-induced sensitivity could be taken into account.

In addition to VSV, VV has been studied as a potential treatment for colon cancer. Bell and colleagues have demonstrated VEGF-induced VV internalization in VECs of mice bearing colon carcinoma tumors. High levels of VEGF were shown to signal through VEGFR2, followed by ERK1/2 and STAT3 signaling, and upregulation of the transcription repressor PRD1/BF1. Thereby, the IFN-mediated anti-viral response of VECs was suppressed, resulting in enhanced sensitivity to VV infection [61].

In addition to these observations of direct virus infection in VECs, OVs have also shown anti-angiogenic effects. Contrast-enhanced ultrasound performed by Thorne and colleagues showed an unexpected rapid vascular collapse and prevention of revascularization upon VV infection in murine breast and renal tumor models. This effect was associated with a VV-induced reduction in VEGF levels during the entire period of viral infection [62]. However, the mechanism underlying the reduced VEGF levels remains to be studied.

Studies focusing on the effect of oncolytic HSV-1 on tumor angiogenesis show contradictory results. While one study has shown a direct anti-angiogenic effect of oncolytic HSV-1 on both murine and human VECs in vitro and in vivo [63], other researchers suggest that HSV-1 could evoke an angiogenic response [64,65]. In human glioma cells, infection with HSV-1-derived OVs increases the angiogenic CYR61 gene expression in a dose-dependent manner in vitro and in vivo. Increased CYR61 expression is linked to glioma vascularization and invasion [64]. Moreover, HSV-1 is shown to have an inherent pro-angiogenic effect by lowering thrombospondin-1 and -2 expression, leading to reduced inhibition of pro-angiogenic factors including VEGF, FGF and MMP9 in infected human glioblastoma and glioma cells in vivo. The mechanism by which HSV-1 inhibits this tumoral expression remains to be studied [64,65]. Notably, human endothelial cells seem susceptible to infection by another virus of the Herpesviridae family, HHV-6, followed by clear anti-angiogenic effects. Upon infection, the expression of HHV-6 U94 has been demonstrated to increase HLA-G expression, which inhibits the angiogenic capacity of the endothelial cells [66]. Altogether, a clear anti- or pro-angiogenic nature of HSV-1 remains to be elucidated.

## 4. Oncolytic Virus Modification to Enhance Stroma Targeting

Despite the natural stroma targeting of the above discussed OVs, the dense tumor stroma can limit the viral spread, replication and infectivity [12]. Stroma targeting by OVs can be enhanced by modifying the OVs to optimize their ability to target either specific stromal components, or their interactions with tumor cells. Below, an overview is given of the different OVs that have been genetically modified, through bioselection or genetic engineering to (better) target specific stromal components (see Table 2).

### 4.1. Modified Oncolytic Viruses Targeting Cancer-Associated Fibroblasts (CAFs)

CAFs represent a promising target for OV therapy as they are the major stromal component. The studies discussed below show how OVs can target CAFs to enhance their replication, or to diminish the pro-tumoral effects and activities of CAFs. To date, they have not examined the targeting of specific CAF subsets. As previously discussed, each CAF subset exerts specific functions. Future studies are warranted to examine how this affects the therapeutic effect of OV-mediated targeting.

A few studies have described bioselection as a powerful tool to select and develop oncolytic adenoviruses with improved stroma-targeting capacities [67]. By repeated passing of a rapidly mutating adenovirus in CAFs, adenovirus mutants that show greater infectivity of these cells have been selected and isolated. After viral genome sequencing, the i-leader C-terminal truncation mutation, iLG397T, was identified to underlie enhanced adenovirus release from both human CAFs and lung tumor cells, as well as cytotoxicity in vitro. The iLG397T mutation was subsequently introduced in two distinctive adenoviruses, resulting in AdiLG397T-RGD [68] and ICOVIR-15i [69]. Upon treatment of xenografts composed of human lung tumor cells and fibroblasts, both iLG397T-mutated adenoviruses augmented the anti-tumor efficacy and animal survival compared to adenoviruses lacking the iLG397T mutation [68,69].

Furthermore, combination treatment with the chemotherapeutic agent gemcitabine in Syrian hamsters bearing syngeneic pancreatic tumors that harbored CAFs was shown to enhance the therapeutic efficacy of ICOVIR-15i. This effect presumably occurs through enhanced tumor cell killing, which may lead to a reduced intratumoral interstitial pressure, followed by enhanced adenoviral penetration. In line with this, ICOVIR-15i-mediated targeting of both CAFs and tumor cells could result in less-dense tumor and stromal tissue, enhancing both OV and gemcitabine penetration [69]. CAF-mediated enhanced virus release remains to be verified in vivo, as the effects have not yet been compared to those in xenografts of tumor cells only. Notably, these studies did not compare the effects to those in xenografts of tumor cells only. Therefore, CAF-mediated enhanced virus release remains to be verified in vivo. In addition, the effects on CAF functions remain unclear, as no specific changes in secretory or cellular functions of CAFs were described.

While bioselection can be a powerful tool for modifying potential OVs, genetic engineering methods have yielded far more of these stroma-targeted OVs. Studies completed by Lopez and colleagues demonstrate the importance of CAF targeting in the oncolytic efficacy of replicative genetically modified adenoviruses. They generated Ad-F512, which expresses a secreted protein acidic and rich in cysteines (SPARC) promoter to selectively replicate in SPARC-expressing cells. SPARC is mainly expressed by stromal cells, including CAFs, and to some extent tumor cells [70]. Ad-F512 infection of xenografts composed of melanoma cells combined with CAF-resembling fibroblasts showed a delay in tumor growth compared to xenografts of only melanoma cells, but no total tumor clearance [70]. Importantly, several epithelial cancer types downregulate SPARC expression by promoter methylation [71], which could hamper the therapeutic efficacy. This highlights the importance of examining SPARC expression levels before considering this approach. The anti-tumor efficacy of Ad-F512 was found to be highly dependent on the presence of fibroblasts. Therefore, Ad-F512 has been further modified, resulting in the strain named Ad-(5/3)-kBF512HRE, in order to improve simultaneous targeting of not only the stromal but also the malignant cell components of the tumors by incorporating hypoxia and inflammation responsive promoter motifs, also described as a triple chimeric promoter [71].

In another approach, the adenovirus strain AdTATMMP has been engineered to selectively enter cells upon proteolytic cleavage of its fiber by MMP2 and MMP9. Since MMP2 and MMP9 are involved in the continued growth of the tumor and metastasis, coupled with the fact that high expression of both is related to poor patient survival in various types of cancers, an OV that targets these markers could be of high clinical value [41]. In comparison to a wild-type fiber adenovirus, AdTATMMP demonstrated increased infection of tumor cells as well as patient-derived CAFs that highly expressed MMP2 and MMP9. OV administration in both syngeneic as well as xenograft models of stroma-rich pancreatic cancer showed a large increase in tumor transduction and tumor killing compared to the wild-type fiber adenovirus [72].

Another way to target CAFs has been exploited with an adenovirus expressing a bispecific T cell engager (BiTe) targeted to FAP. FAP, as previously discussed, is one of the general markers expressed by CAFs. This virus, EnAd-CMV-FAP-BiTE, was shown to trigger T cell-mediated killing of CAFs in human malignant ascites as well as solid prostate cancer tissue [73]. A similar adenovirus, ICO15K-FBiTE, encodes a FAP-targeting BiTE as well. ICO15K-FBiTE treatment of mice bearing human lung or pancreatic tumors stimulated T cell activation and T cell infiltration into tumors, which mediated killing of FAP-positive cells, and reduced tumor growth. This indicates that this virus, like EnAd-FAP-BiTE, stimulates T cell-mediated killing of FAP-expressing CAFs [74].

CAFs have also been targeted using a genetically modified measles virus (MV) that is retargeted against the murine or human uPAR (MV-m-uPA or MV-h-uPA, respectively) [44]. The MV-uPA viruses were shown to efficiently infect and kill murine and human CAFs. These viruses were also effective against breast tumor cells in a species-specific manner in vitro and in vivo. It was demonstrated that MV-uPA could be successfully transferred from infected fibroblasts to tumor cells. Murine CAFs infected by murine MV-uPA inhibited the proliferation of human breast cancer cells that were otherwise MV-m-UPA resistant, whereas uninfected CAFs stimulated tumor cell growth. Moreover, the importance of stromal targeting was demonstrated in a murine xenograft model containing human breast tumor cells (MV-m-uPA resistant, MV-h-uPA sensitive) and murine stromal cells (MV-m-uPA sensitive, MV-h-uPA resistant). Whereas administration of MV-h-uPA showed superior tumor-controlling effects over MV-m-uPA, a combination treatment of both MV-m-uPA and MV-h-uPA outperformed monotherapy [44].

In a recent follow-up study, MV-CD46-muPA has been developed, which was shown to target both human CD46-positive colon tumor cells and murine uPAR-expressing CAFs in vitro and in vivo. Systemic administration of MV-CD46-muPA to mice bearing human colon tumors improved tumor cell killing and overall survival compared to MV-GFP, indicating the importance of killing both tumor and stromal cells. Notably, the effects of MV-CD46-muPA were not compared with those of MV-m-uPA, MV-h-uPA, or combined MV-m-uPA/MV-h-uPA treatment [65]. Another study by the same group has demonstrated targeting of uPAR-expressing endothelium cells by MV-uPA, which is further discussed below. The effects on other uPAR-expressing cells such as TAMs remain to be studied.

Another way to target CAFs has been shown using genetically modified recombinant vaccinia virus, known as GLV-1h282, that expresses FAP-specific single-chain antibody (scAB). Upon intravenous injection of mice bearing human lung epithelial tumor xenografts, GLV-1h282 has demonstrated enhanced tumor regression compared to the unmodified GLV-1h68. This regression was likely related to OV targeting of FAP-expressing CAFs present in the model, as the tumor cells were shown to be FAP negative. Interestingly, the greatest tumor regression was shown after treatment with the engineered vaccinia viruses encoding scABs targeting both VEGF and EGFR (GLV-1h444), or VEGF and FAP (GLV-1h446). Although no direct CAF targeting was described, the observed tumor growth inhibition using GLV-1h446 was presumed to be partially attributed to CAF targeting [75].

Altogether, these studies highlight the potential of generating OVs with enhanced stroma-targeting capacities through bioselection and genetic engineering. Importantly, the composition of CAF subsets and functions differ between tumors, as mentioned before. A description of the specific human CAF subsets involved in the experiments is lacking, rendering it challenging to extrapolate the findings to other tumors.

#### Potential Approaches to Target Pro-Tumoral Cancer-Associated Fibroblast Subsets (CAF Subsets)

It has become clear that genetically engineered OVs are able to target CAFs, which can improve OV distribution, infectivity in the tumor stroma, and therapeutic outcome in a variety of tumor types. However, the performed studies lack a clear CAF subset description, as for example FAP is a general CAF marker for both anti-tumoral myCAFs and pro-tumoral iCAFs [45]. Moreover, targeting of non-specific CAF markers demands caution, as this could result in undesirable off-targeted effects on other cell types expressing the markers [37]. A more detailed examination of the specific CAF populations to be targeted for distinct tumor types, based on surface markers and secretory phenotype, would greatly benefit the design of robust and consistent CAF-targeting OV therapies. Below, some recommendations are given on strategies to further improve CAF (subset) targeting.

The recently identified pro-tumoral iCAFs represent a favorable target for OV therapy. In order to specifically target iCAFs, OVs should be directed against one of the specific iCAF markers (Table 1). AGTR1 in particular poses a promising target, as this surface marker is already studied in current clinical trials [48,49]. Moreover, iCAF formation could be inhibited by blocking the IL-1α-induced NF-κB-LIF/JAK/STAT signaling pathway in fibroblasts, e.g., using IL-1 receptor antagonists or IL-1α neutralizing antibodies [47,76]. This approach represents a double-edged sword, reducing the number of pro-tumorigenic iCAFs [47] and shifting the iCAF phenotype towards the tumor-restraining myCAF phenotype [36]. Along the same line, inducing the TGF-β signaling pathway, which presumably underlies the function of myCAFs, could increase the number of tumor-restraining myCAFs [47]. Alternatively, OVs could be engineered to express recombinant sperm hyaluronidase or hyaluronan acid synthesis inhibitors to deplete the iCAF-secreted hyaluronan synthases (Table 1). The hyaluronan-induced impairment of the tumor vasculature would then be diminished, enhancing drug delivery and OV spread in solid tumors [48].

The pro-tumoral CD10+GPR77+ CAFs pose another interesting CAF subset for future OV therapy. GPR77, a surface marker and an essential signaling molecule for this specific CAF subset (Table 1) [50], could be targeted by OVs through the expression of, e.g., GPR77-neutralizing antibodies. Overall, there are various potential OVs targeting these different CAFs that could be developed in future studies.

### 4.2. Engineered Oncolytic Viruses Targeting the Tumor Extracellular Matrix (ECM)

OV-expressed factors such as proteases can be used to degrade ECM components. For example, MMP9 has been shown to facilitate ECM degradation, and to enhance HSV delivery, distribution, and oncolytic effect in mice bearing human brain tumors [77]. Recently, Grandi and colleagues armed an EGFR-retargeted HSV with MMP9, KMMP9. This modified HSV demonstrated selective infection and killing of EGFR-bearing glioblastoma tumor cells in vitro and improved viral penetration and survival in a glioblastoma multi-forme xenograft model [78]. Whether this HSV-mediated ECM degradation also increases (anti-tumor) immune cell infiltration in the tumor remains to be studied. Similarly, Szalay and colleagues showed that intratumoral administration of GLV-1h255, a VV expressing MMP9, in mice bearing prostate tumors enhanced collagen IV degradation, viral replication, and tumor regression [79]. Strikingly, MMP9 has been found to induce VEGF bioavailability, leading to enhanced angiogenesis [80], highlighting a potential undesirable effect of MMP9 expression by OVs. While Szalay and colleagues disproved this, as the VV-infected areas of the prostate tumor showed significantly lower vascular density compared to uninfected areas [79], Grandi and colleagues did not study this possible adverse effect in their experiments [78].

Conversely, MMP suppression has been exploited to counteract the pro-tumorigenic roles of MMPs. Tissue inhibitor metalloproteinases 1 to 4 (TIMPs1-4) tightly regulate the proteolytic activity of MMPs in healthy human tissues. A decreased expression of TIMPs, and therefore over-active MMPs, has been shown to result in invasive, metastatic, and poor prognostic tumors [81]. Therefore, Cripe and colleagues engineered an oncolytic TIMP-3-armed HSV, rQT3, in order to neutralize MMP3 expression. TIMP-3-armed HSV treatment of human neural tumor cells in vitro and in vivo inhibited MMP3 levels, increased viral replication in and cytotoxicity of tumor cells as well as VECs, thereby reducing vascular density [82].

Altogether, targeting the ECM requires caution. The efficacy of either MMP expression or suppression to promote OV spread in the tumor stroma may depend on the tumor-specific ECM composition, requires adequate dosing and timing, and should be weighed against the potential release of tumor-promoting factors [81]. MMP expression by OVs is another potential concern, as the MMP-mediated ECM remodeling is also known to facilitate tumor growth, tumor cell invasion, and metastasis [83]. In addition to this pro-tumorigenic effect, some MMPs can also exert anti-viral activity on OVs [84]. Moreover, the TIMP-3, MMPs and tumor growth factors present in the ECM use heparan sulfate proteoglycans as docking sites. In addition, MMPs are described to cleave many non-matrix substrates including cellular receptors, which together could interfere with the OV binding [85]. To date, these effects have not been addressed in studies on the effectiveness of such approaches. These interactions are likely more apparent in the complex 3D tumor tissues in comparison with the dissimilar two-dimensional cell cultures. Therefore, it remains to be determined whether the mentioned MMP-targeting approaches are also effective in the clinic.

### 4.3. Engineered Oncolytic Viruses Targeting the Tumor Vasculature

Multiple approaches exist to target the tumor vasculature using genetically engineered OVs [86]. These approaches could be divided into OVs targeting the tumor vasculature by infecting the tumor VECs or inhibiting (pathways of) angiogenic cytokines and proteins specifically.

Firstly, as previously discussed for CAFs, a variant of the SPARC promotor expressing Ad-F512, Ad(I)-F512-TK, is shown to effectively infect and kill both CAFs and endothelial cells in vitro. Combination treatment of this OV with gemcitabine of mice bearing a mix of pancreatic tumor cells and endothelial cells showed induced complete tumor remission compared to mice bearing tumor cells only [70]. This implies that the presence of the endothelial cells favored the therapeutic efficacy of this combination treatment. The effect of Ad(I)-F512-TK on tumor cells mixed with CAFs in vivo remains to be elucidated.

Another OV which has also been shown to target VECs, in addition to CAFs, is the earlier discussed MV-uPA. Compared to MV-GFP, MV-m-uPA enhances endothelial cell infection both in vitro and in systemically infected mice bearing human breast tumors. Moreover, MV-m-uPA treatment resulted in superior anti-tumor effect and prolongation of survival compared to mock infection. No toxicity was observed, indicating specific targeting of uPAR-expressing endothelial cells [87]. Altogether, it is proven that both CAFs and endothelial cells can be efficiently targeted by OVs based on their SPARC and uPAR expression. However, to date, no in vivo model has been described in which the effect of OV therapy on tumor mixed with both stromal components is studied. Therefore, additional studies are needed to prove whether such multi-faceted OV therapies are more efficacious than single-targeted ones.

The oncolytic bG47Delta-dnFGFR, an HSV expressing a FGFR therapeutic transgene, is shown to be another approach to inhibit the tumor vasculature. Upon treatment with this OV, mice bearing human glioblastoma tumors showed both angiogenesis and tumor growth inhibition. These results could be related to inhibition of FGF signaling in both VECs and tumor cells [88]. Importantly, FGF2 is overexpressed in both human head and neck squamous cell carcinoma (HNSCC) and VECs, promoting tumor vascularization [89]. The effect of this FGF2 overexpression can be diminished using FGF2-TK, an adenovirus carrying recombinant FGF2-Fab. Treating both glioblastoma and HNSCC tumors using this OV is shown to reduce tumor vascularization and cell growth in murine xenografts [89].

Another way to target VECs, currently studied in clinical trials, is the multi-functional engineered JX-594, an oncolytic VV with a disrupted viral thymidine kinase (TK) gene [90]. JX-594 therefore requires a higher level of cellular TK for its replication, compared to TK-positive wild-type VV. In addition, as previously discussed, VV takes over cellular pathways such as VEGF-induced ERK1/2 and STAT3 signaling for its replication in VECs [61]. VEGF stimulation was shown to stimulate VEGFR phosphorylation in endothelial cells, which resulted in TK expression and enhanced JX-594 replication and release. These results were in line with the observed tumor and tumor vasculature disruption in JX-594-infected mice bearing breast tumors that expressed high levels of VEGF and in hypervascular liver cancer patients [90].

OVs have also shown anti-vascular effects by decreasing pro-angiogenic cytokines or proteins in tumor tissues. One approach is to target the highly expressed MMP14 in human glioma cells using an oncolytic adenovirus encoding an MMP14-silencing small hairpin RNA (shRNA), CRAd-S-5/3shMMP14. MMP14 expression is known for its angiogenesis-promoting effect, therefore silencing of this protein is shown to reduce pro-angiogenic IL-8 and VEGF levels in infected glioma cells in vitro, as well as angiogenesis and glioma proliferation in human glioma xenografts [91].

Another adenovirus has been engineered to carry the anti-sense sequence for uPAR and MMP9, Ad-uPAR-MMP9. MMP9 is a collagen type IV-degrading enzyme, by which it contributes to metastasis, as well as an effective diagnostic biomarker for non-small-cell lung cancer (NSCLC) [92]. Ad-uPAR-MMP9 infection was shown to downregulate uPAR and MMP9 protein levels and inhibit angiogenesis in human lung tumor cells in vitro. Moreover, human NSCLC-bearing mice showed higher percentages (90%) of tumor control and distinct inhibition of metastasis upon infection with Ad-uPAR-MMP9 compared to unarmed adenovirus, which was attributed to adenovirus-mediated destruction of the tumor vasculature [93].

An additional set of approaches involve engineered OVs targeting pro-angiogenic cytokines in order to target the tumor vasculature. Firstly, a study of Hemminki and colleagues has shown a clear anti-angiogenic effect using vvdd-VEGFR-1-Ig, an engineered VV targeted against kidney tumor cells that is armed with VEGFR-1-Ig. This VEGFR-1 is known to scavenge the highly expressed VEGF molecules, inhibiting VEGF-induced angiogenesis. Upon medium or low doses of systemic OV administration in renal tumors of immunodeficient mice, tumor regression, anti-tumor cytokine responses and anti-angiogenic effects were detected [94].

In line with this, infection with the earlier introduced VVs encoding antibodies that target VEGF and EGFR (GLV-1h44), or VEGF and FAP (GLV-1h446) diminished angiogenesis and tumor cell proliferation in human prostate tumor xenografts. GLV-1h446, expressing anti-VEGF and anti-FAP, was specifically shown to decrease tumor proliferation in both infected and uninfected distal tumor areas, demonstrating the enhanced systemic therapeutic effects of this VV [75]. Interestingly, the previously discussed natural VECs tropism of VVs is facilitated by VEGF signaling which could cause a counter effect of inhibiting VEGF-signaling on the natural CAF tropism of VV [59]. This possible counter effect of VVs has not yet been examined by either of the above discussed studies.

IL-12 has been demonstrated to enhance Th1-mediated anti-tumor responses and to inhibit tumor angiogenesis in severe-combined immunodeficient mice [95]. This has been exploited by the engineering of an IL-12-armed HSV, NV1042 [96]. Combined treatment of this virus and a low dose of vinblastine, a microtubule-disrupting agent, was shown to target prostate tumor cells and reduce angiogenesis by impairing VEC growth, neovascularization, and tumorigenesis in a murine xenograft model [96].

Finally, the tumor vasculature has also been targeted by OVV-CXCR4-A-mFc, a VV expressing a CXCR4 antagonist, which is directed against the VEGF-induced pro-angiogenic CXCL12/CXCR4 signaling pathway in human breast tumor cells. Upon intravenous administration, this virus blocked CXCL12/CXCR4 signaling, inhibiting the recruitment of circulating endothelial progenitors (CEPs) into the stroma and destructing the tumor vasculature in human breast tumor bearing mice [97].

### 4.4. Engineered Oncolytic Virus Indirect Targeting of Tumor-Associated Macrophages (TAMs)

Targeting of the highly abundant TAM population and their interactions with the tumor cells and other stromal components may help to reduce primary tumor growth, metastases, and recurrence. Multiple therapeutic approaches to reduce the pro-tumoral effects of TAMs have been reviewed by Portella and colleagues, including TAM depletion, reduction in macrophage recruitment, or modulation of the pro-tumoral M1 to M2 macrophages polarization [54]. The oncolytic effect of OVs is shown to be closely related to the macrophage phenotype present in the tumor stroma. While M1 macrophages enhance viral clearance, they also reduce tumor growth and can therefore increase the oncolytic effect of OVs. In contrast, despite the anti-inflammatory effect, and thereby the prevention of viral clearance, the above discussed pro-tumoral effects of M2 phenotypes counteract the oncolytic effect of OVs. Therefore, a growing body of studies describes the effect of OV therapies and the indirect immune-modulatory effects, of which the pro-tumoral effects of TAMs are shown to be reduced as result of either enhanced infiltration or repolarization of the M1 phenotype rather than M2 phenotype [44,54].

Despite the importance of TAMs in steering the pro-tumoral and immunosuppressive environment of stromal tissues, no (engineered) OVs have been documented yet in which TAMs are directly targeted by OVs. Taken together, future studies focusing on OVs targeting TAMs by, for example, expressing transgenes directed against the previously discussed TAM markers such as CD163, CD204 or CD200R could be of great value.

## 5. Conclusion and Future Outlooks

OVs represent a promising multi-faceted class of tumor therapeutics to overcome the dense tumor stroma (Figure 1). Excluding lymphocytes such as Tregs, the major tumor stromal components include CAFs, VECs, TAMs, and ECM proteins such as MMPs. Several OVs have shown an inherent capability to naturally target CAFs and VECs. However, the dense stroma often limits OV spread, replication and infectivity. Therefore, multiple approaches have been described to optimize targeting of both tumor and stromal components by using genetically engineered OVs. Using these approaches, OV-mediated stromal targeting has been shown to efficiently increase tumor control and overall survival.

By targeting the rich ECM and its components such as MMPs, the density and stiffness of the tumor ECM will presumably decrease, easing OV, anti-tumor drug and lymphocyte migration towards the tumor [51]. The subsequent cascade of immunological events are beyond the scope of this article, but have been excellently reviewed by others [18,51]. Importantly, angiogenesis inhibition is anticipated to reduce tumor vasculature, but also to decrease the recruitment of immune cells via the bloodstream and thereby suppress anti-tumor immune responses [98].

In particular, OV-mediated targeting of CAFs appears promising, as CAFs are known as the ‘small factories’ of the dense ECM. In addition, CAFs have been identified as the major players in maintaining a pro-tumoral niche as well as regulating the complex tumor–stroma interactions. Multiple non-specific CAF markers have been identified. However, the heterogeneity of CAF subsets and their tumor-distinctive functions in different tumor types highlight the importance of examining and targeting more specific CAF populations that may differ per tumor tissue. The best-studied CAF subsets are the myCAFs and iCAFs present in pancreatic tumors. Future OVs should preferably be engineered to target the pro-tumorigenic iCAFs or CD10+GPR77+ CAFs by targeting specific surface markers, or the signaling pathways involved in their formation and function.

Importantly, stroma targeting by OVs demands some caution, as it could have major effects on the tumor composition, immune infiltration and activation, as well as off-target effects on surrounding cells. Altogether, by taking into account the complex interactions between the heterogenous stroma components and tumor cells, OVs and their arming transgenes hold promise as unique dual tumor- and stroma-targeted therapies that could ultimately improve the prognosis of cancer patients, especially those suffering from stroma-rich tumors.

## Figures and Tables

**Table 1:** Characteristics and functions of five distinctive CAF subsets.

CAF Subset	Effect on Tumor		Characteristics		
myCAFs	Anti-tumoral (pancreatic tumor) [36,37,38]	Expression of COL1A1, FAP, VIM	↓ Expression of co-stimulatory molecules CD80, CD86, CD40	Expression of PDPN, DCN	↑ Expression of αSMA, TAGLN, MYL9, TPM1/2, MMP11, POSTN, and HOPX
					↑ Collagen production
					Restrains tumor upon Hedgehog activation
					Formation upon TGFβ-SMAD2/3 signaling
iCAFs	Pro-tumoral (pancreatic tumor) [36,38,39,40]				↑ Expression of AGTR1, PDGFRA, CFD, LMNA, and DPT
					Secretion of inflammatory mediators, e.g., IL-6, G-CSF, LIF, CXCL1
					Hyaluronan synthesis→ ↑ migration/invasion tumor cells→ impaired vasculature → therapeutic resistance
					Formation upon IL-1α induced NF-κB and LIF/JAK-STAT3 signaling
apCAFs	Pro-tumoral (pancreatic tumor) [36]				Expression of MHC-II and H2-Ab1
					Present antigens to CD4+ T cells, lack co-stimulatory molecules → hypothetically deactivation by inducing anergy or differentiation into Tregs
cCAFs	Pro-tumoral (breast and prostate tumors) [39]				Circulate individually or clustered with tumor cells
					Metastasis and tumor cell colonization
CD10+GPR77+ CAFs	Pro-tumoral (breast and lung tumors) [41]				CD10 and GPR77
					↑ NF-kB activation → IL-6, IL-8 secretion → cancer stemness and persistence

↑, upregulation; ↓, downregulation; →, leading to; CAF, cancer-associated fibroblasts; myCAFs, myofibroblastic CAFs; iCAFs, inflammatory CAFs; apCAFs, antigen-presenting CAFs; cCAFs, circulating CAFs; GPR77, G-protein-coupled receptor 77; COL1A1, collagen type I alpha I chain; FAP, fibroblast-associated protein; VIM, vimentin; PDPN, podoplanin; DCN, decorin; αSMA, α-smooth muscle actin; TAGLN, transgelin; MYL9, myosin light chain 9; TPM1/2, tropomyosins 1 and 2; MMP11, matrix metallopeptidase 11; POSTN, periostin; HOPX, homeobox transcription factor; TGFβ, transforming growth factor beta; AGTR1, angiotensin II receptor type 1; PDGFRA, platelet-derived growth factor receptor alpha; CFD, complement factor D; LMNA, laminin A/C; DPT, dermatopontin; IL-6, interleukin-6; G-CSF; granulocyte-colony stimulating factor; CXCL1, chemokine C-X-C motif ligand 1; IL-1α, interleukin-1 alpha; LIF, leukemia inhibitory factor; JAK/STAT3, janus kinase/signal transducer and activator of transcription-3; MHC-II, major histocompatibility complex class II; H2Aa/b1, histocompatibility 2 class II antigen A alpha and beta1; NF-kB, nuclear factor kappa-light-chain-enhancer of activated B cells; IL-8, interleukin-8.

**Table 2:** Overview of inherent, bioselected and genetically engineered OVs targeting tumor stroma components.

Virus/Vector	Modification/Transgene	Stroma Target	Observations
*Natural targeting*			
VSV		CAFs, vasculature [49,51]	CAF and VEC infection
Reovirus		Vasculature [50]	Viral replication in VECs
VV		Vasculature [52,53]	↓ Anti-viral response VECs; ↓ (Re)vascularization
HSV-1		Vasculature [54,55,56]	Anti-/pro-angiogenic effects
HHV-6		Vasculature [57]	↓ Angiogenesis
*Bioselected for enhanced targeting*			
AdiLG397T-RGD	iLG39T mutation	CAFs [59]	CAF infection and killing
*Genetically modified for enhanced targeting*			
Ad-F512, -TK/GCV	SPARC promoter	CAFs, vasculature [61]	CAF and VEC killing
Ad-(5/3)-kBF512HRE	Triple chimeric promoter	CAFs [62]	CAF killing
AdTATMMP	MMP-cleavable linker	CAFs [63]	↑ CAF infection, ↑ killing
EnAd-CMV-FAP-BiTE	FAP-targeted BiTE	CAFs [64]	T cell-mediated CAF killing
ICO15k-FBiTE	FAP-targeted BiTE	CAFs [65]	T cell-mediated CAF killing
MV-m/h-uPA	uPA	CAFs; vasculature [35,78]	Target uPAR^+^ CAFs and VECs
GLV-1h282	Anti-FAP	CAFs [66]	CAF killing
KMMP9	EGFR, MMP9	ECM [69]	↓ ECM → ↑ HSV penetration
GLV-1h255	MMP9	ECM [70]	↓ ECM → ↑ replication
rQT3	TIMP-3	ECM [73]	↓ Tumor (several hypotheses)
bG47Delta-dnFGFR	FGFR	Vasculature [79]	↑ VEC killing
FGF2-TK	FGF2-Fab	Vasculature [80]	Targets FGF2^+^ VECs
JX-594	TK disrupted	Vasculature [81]	Targets TK^+^ VECs
CRAd-S-5/3shMMP14	MMP14 shRNA	ECM [82]	↓ Angiogenesis
Ad-uPAR-MMP9	Anti-sense uPAR and MMP9	ECM [84]	↓ Angiogenesis
vvdd-VEGFR-1-Ig	VEGFR-1	Vasculature [85]	↓ Angiogenesis
GLV-1h444	Anti-VEGF/-EGFR	Vasculature [66]	↓ Blood vessel density
GLV-1h446	Anti-EGFR/-FAP	Vasculature, CAFs [66]	↓ Blood vessel density, likely CAF targeting
NV1042	IL-12	Vasculature [87]	↓ Neovascularization
OVV-CXCR4-A-mFc	CXCR4 antagonist	Vasculature [88]	↓ Angiogenesis

↑, upregulation; ↓, downregulation; →, leading to; VSV, vesicular stomatitis virus; VV, vaccinia virus; HSV, herpes simplex virus; HHV, human herpesvirus; Ad, adenovirus; MV, measles virus. TK, thymidine kinase; GCV, ganciclovir; MMP, matrix metalloprotease; FAP, fibroblast activation protein; BiTE, bispecific T cell engager; uPA(R), urokinase-type plasminogen activator (receptor); FAP, fibroblast activation protein; EGFR, epidermal growth factor receptor; TIMP, tissue inhibitor metalloproteinase; FGF(R), fibroblast growth factor (receptor); shRNA, short hairpin RNA; VEGFR, vascular-endothelial growth factor receptor; IL, interleukin; CXCR4, chemokine C-X-C motif receptor 4; CAF, cancer-associated fibroblasts; VECs, vascular endothelial cells; ECM, extracellular matrix.

## References

[1] (2018). Mortality Database Health Statistics and Information Systems.

[2] Topalian S.L., Hodi F.S., Brahmer J.R., Gettinger S.N., Smith D.C., McDermott D.F., Powderly J.D., Carvajal R.D., Sosman J.A., Atkins M.B. (2012). Safety, activity, and immune correlates of anti-PD-1 antibody in cancer. N. Engl. J. Med..

[3] Kather J.N., Halama N., Jaeger D. (2018). Genomics and emerging biomarkers for immunotherapy of colorectal cancer. Semin. Cancer Biol..

[4] Iivanainen S., Alanko T., Peltola K., Konkola T., Ekström J., Virtanen H., Koivunen J.P. (2019). ePROs in the follow-up of cancer patients treated with immune checkpoint inhibitors: A retrospective study. J. Cancer Res. Clin. Oncol..

[5] Schirrmacher V. (2019). From chemotherapy to biological therapy: A review of novel concepts to reduce the side effects of systemic cancer treatment (Review). Int. J. Oncol..

[6] Gegechkori N., Haines L., Lin J.J. (2017). Long-Term and Latent Side Effects of Specific Cancer Types. Med. Clin. N. Am..

[7] Ramos P., Bentires-Alj M. (2015). Mechanism-based cancer therapy: Resistance to therapy, therapy for resistance. Oncogene.

[8] Laboratory C.S.H. (2017). Why Is Pancreatic Cancer So Hard to Treat? Stroma Provides New Clues. ScienceDaily.

[9] Kenneth C.V., de groot A.E., Kenneth C.P. (2018). Targeting the tumour stroma to improve cancer therapy. Nat. Rev. Clin. Oncol..

[10] Seager R.J., Hajal C., Spill F., Kamm R.D., Zaman M.H. (2017). Dynamic interplay between tumour, stroma and immune system can drive or prevent tumour progression. Converg. Sci. Phys. Oncol..

[11] Stephen J.R., Kah-Whye P., John C.B. (2014). Oncolytic virotherapy. Nat. Biotechnol..

[12] Harrington K., Freeman D.J., Kelly B., Harper J., Soria J.C. (2019). Optimizing oncolytic virotherapy in cancer treatment. Nat. Rev. Drug Discov..

[13] Achard C., Surendran A., Wedge M.E., Ungerechts G., Bell J., Ilkow C.S. (2018). Lighting a Fire in the Tumor Microenvironment Using Oncolytic Immunotherapy. EBioMedicine.

[14] Jhawar S.R., Thandoni A., Bommareddy P.K., Hassan S., Kohlhapp F.J., Goyal S., Schenkel J.M., Silk A.W., Zloza A. (2017). Oncolytic viruses-natural and genetically engineered cancer immunotherapies. Front. Oncol..

[15] Gulley J.L., Madan R.A., Pachynski R., Mulders P., Sheikh N.A., Trager J., Drake C.G. (2017). Role of antigen spread and distinctive characteristics of immunotherapy in cancer treatment. J. Natl. Cancer Inst..

[16] Raja J., Ludwig J.M., Gettinger S.N., Schalper K.A., Kim H.S. (2018). Oncolytic virus immunotherapy: future prospects for oncology. J. Immunother. Cancer.

[17] Lin C.Z., Xiang G.L., Zhu X.H., Xiu L.L., Sun J.X., Zhang X.Y. (2018). Advances in the mechanisms of action of cancer-targeting oncolytic viruses (review). Oncol. Lett..

[18] de Graaf J.F., de Vor L., Fouchier R.A.M., van den Hoogen B.G. (2018). Armed oncolytic viruses: A kick-start for anti-tumor immunity. Cytokine Growth Factor Rev..

[19] Fiola C., Peeters B., Fournier P., Arnold A., Bucur M., Schirrmacher V. (2006). Tumor selective replication of Newcastle Disease Virus: Association with defects of tumor cells in antiviral defence. Int. J. Cancer.

[20] Eric B., Grant M. (2009). Human cancer cells have specifically lost the ability to induce the synergistic state caused by tumor necrosis factor plus interferon-beta. Cytokine.

[21] Stojdl D.F., Lichty B., Knowles S., Marius R., Atkins H., Sonenberg N., Bell J.C. (2000). Exploiting tumor-specific defects in the interferon pathway with a previously unknown oncolytic virus. Nat. Med..

[22] Munir M., Berg M. (2013). The multiple faces of proteinkinase R in antiviral defense. Virulence.

[23] Eissa I.R., Bustos-Villalobos I., Ichinose T., Matsumura S., Naoe Y., Miyajima N., Morimoto D., Mukoyama N., Zhiwen W., Tanaka M. (2018). The current status and future prospects of oncolytic viruses in clinical trials against melanoma, glioma, pancreatic, and breast cancers. Cancers.

[24] Zheng M., Huang J., Tong A., Yang H. (2019). Oncolytic Viruses for Cancer Therapy: Barriers and Recent Advances. Mol. Ther. Oncolytics.

[25] Leber M.F., Neault S., Jirovec E., Barkley R., Said A., Bell J.C., Ungerechts G. (2020). Engineering and combining oncolytic measles virus for cancer therapy. Cytokine Growth Factor Rev..

[26] Munis A.M., Bentley E.M., Takeuchi Y. (2020). A tool with many applications: vesicular stomatitis virus in research and medicine. Expert Opin. Biol. Ther..

[27] Bourhill T., Mori Y., Rancourt D.E., Shmulevitz M., Johnston R.N. (2018). Going (Reo)viral: Factors promoting successful reoviral oncolytic infection. Viruses.

[28] Fu L.Q., Wang S.B., Cai M.H., Wang X.J., Chen J.Y., Tong X.M., Chen X.Y., Mou X.Z. (2019). Recent advances in oncolytic virus-based cancer therapy. Virus Res..

[29] Cuadrado-Castano S., Sanchez-Aparicio M.T., García-Sastre A., Villar E. (2015). The therapeutic effect of death: Newcastle disease virus and its antitumor potential. Virus Res..

[30] Song H., Zhong L.-P., He J., Huang Y., Zhao Y.-X. (2019). Application of Newcastle disease virus in the treatment of colorectal cancer. World J. Clin. Cases.

[31] Sato-Dahlman M., Larocca C.J., Yanagiba C., Yamamoto M. (2020). Adenovirus and immunotherapy: Advancing cancer treatment by combination. Cancers.

[32] Torres-Domínguez L.E., McFadden G. (2019). Poxvirus oncolytic virotherapy. Expert Opin. Biol. Ther..

[33] (2020). Rahman; McFadden Oncolytic Virotherapy with Myxoma Virus. J. Clin. Med..

[34] Spill F., Reynolds D.S., Kamm R.D., Zaman M.H. (2016). Impact of the physical microenvironment on tumor progression and metastasis. Curr. Opin. Biotechnol..

[35] Santi A., Kugeratski F.G., Zanivan S. (2018). Cancer Associated Fibroblasts: The Architects of Stroma Remodeling. Proteomics.

[36] Vähä-Koskela M., Hinkkanen A. (2014). Tumor restrictions to oncolytic virus. Biomedicines.

[37] Chen X., Song E. (2019). Turning foes to friends: targeting cancer-associated fibroblasts. Nat. Rev. Drug Discov..

[38] Sahai E., Astsaturov I., Cukierman E., DeNardo D.G., Egeblad M., Evans R.M., Fearon D., Greten F.R., Hingorani S.R., Hunter T. (2020). A framework for advancing our understanding of cancer-associated fibroblasts. Nat. Rev. Cancer.

[39] Awaji M., Futakuchi M., Heavican T., Iqbal J., Singh R.K. (2019). Cancer-Associated Fibroblasts Enhance Survival and Progression of the Aggressive Pancreatic Tumor Via FGF-2 and CXCL8. Cancer Microenviron..

[40] Denton A.E., Roberts E.W., Fearon D.T. (2018). Stromal Cells in the Tumor Microenvironment. Stromal Immunology.

[41] Liu T., Zhou L., Li D., Andl T., Zhang Y. (2019). Cancer-associated fibroblasts build and secure the tumor microenvironment. Front. Cell Dev. Biol..

[42] Monteran L., Erez N. (2019). The dark side of fibroblasts: Cancer-associated fibroblasts as mediators of immunosuppression in the tumor microenvironment. Front. Immunol..

[43] Kalluri R. (2016). The biology and function of fibroblasts in cancer. Nat. Rev. Cancer.

[44] Yuqi J., Valery C., Yuguang B., Nicolas A., Doraya E.-A., Alexey P., Xi C., Jaime R M. (2017). Molecular Effects of Stromal Selective Targeting by uPAR Retargeted Oncolytic Virus in Breast Cancer. Mol. Cancer Res..

[45] Elyada E., Bolisetty M., Laise P., Flynn W., Courtois E., Bukhart R., Teinor J., Belleau P., Tuveson D. (2019). Cross-species single-cell analysis of pancreatic ductal adenocarcinoma reveals antigen-presenting cancer-associated fibroblasts. Cancer Discov..

[46] Rhim A.D., Oberstein P.E., Thomas D.H., Mirek E.T., Palermo F., Sastra S.A., Dekleva E.N., Saunders T., Becerra C.P., Tattersall I.W. (2014). Stromal elements act to retrain rather than support pdac. Cancer Cell.

[47] Biffi G., Oni T.E., Spielman B., Hao Y., Elyada E., Park Y., Preall J., Tuveson D.A. (2019). IL1-Induced JAK/STAT Signaling Is Antagonized by TGFβ to Shape CAF Heterogeneity in Pancreatic Ductal Adenocarcinoma. Cacer Discov..

[48] McCarthy J.B., El-Ashry D., Turley E.A. (2018). Hyaluronan, cancer-associated fibroblasts and the tumor microenvironment in malignant progression. Front. Cell Dev. Biol..

[49] Jacobetz M.A., Chan D.S., Neesse A., Bapiro T.E., Cook N., Frese K.K., Feig C., Nakagawa T., Caldwell M.E., Zecchini H.I. (2013). Hyaluronan impairs vascular function and drug delivery in a mouse model of pancreatic cancer. Gut.

[50] Su S., Chen J., Yao H., Liu J., Yu S., Lao L., Wang M., Luo M., Xing Y., Chen F. (2018). CD10+GPR77+ Cancer-Associated Fibroblasts Promote Cancer Formation and Chemoresistance by Sustaining Cancer Stemness. Cell.

[51] Henke E., Nandigama R., Ergün S. (2020). Extracellular Matrix in the Tumor Microenvironment and Its Impact on Cancer Therapy. Front. Mol. Biosci..

[52] Choi H., Moon A. (2018). Crosstalk between cancer cells and endothelial cells: implications for tumor progression and intervention. Arch. Pharm. Res..

[53] Maishi N., Hida K. (2017). Tumor endothelial cells accelerate tumor metastasis. Cancer Sci..

[54] Malfitano A.M., Pisanti S., Napolitano F., Di Somma S., Martinelli R., Portella G. (2020). Tumor-associated Macrophage Status in Cancer Treatment. Cancers.

[55] Jayasingam S.D., Citartan M., Thang T.H. (2020). Evaluating the Polarization of Tumor-Associated Macrophages Into M1 and M2 Phenotypes in Human Cancer Tissue: Technicalities and Challenges in Routine Clinical Practice. Front. Oncol..

[56] Mantovani A., Marchesi F., Malesci A., Laghi L. (2017). Tumour-associated macrophages as treatment targets in oncology. Nat. Rev. Clin. Oncol..

[57] Larionova I., Cherdyntseva N., Liu T. (2019). Interaction of tumor-associated macrophages and cancer chemotherapy. Oncoimmunology.

[58] Ilkow C.S., Marguerie M., Batenchuk C., Mayer J., Ben Neriah D., Cousineau S., Falls T., Jennings V.A., Boileau M., Bellamy D. (2015). Reciprocal cellular cross-talk within the tumor microenvironment promotes oncolytic virus activity. Nat. Med..

[59] Kottke T., Hall G., Pulido J., Diaz R.M., Thompson J., Chong H., Coffey M., Pandha H., Chester J., Melcher A. (2010). Antiangiogenic cancer therapy combined with oncolytic virotherapy leads to regression of established tumors in mice. Cancer Res..

[60] Breitbach C.J., De Silva N.S., Falls T.J., Aladl U., Evgin L., Paterson J., Sun Y.Y., Roy D.G., Rintoul J.L., Daneshmand M. (2011). Targeting tumor vasculature with an oncolytic virus. Mol. Ther..

[61] Arulanandam R., Batenchuk C., Angarita F.A., Ottolino-Perry K., Cousineau S., Mottashed A., Burgess E., Falls T.J., De Silva N., Tsang J. (2015). VEGF-Mediated Induction of PRD1-BF1/Blimp1 Expression Sensitizes Tumor Vasculature to Oncolytic Virus Infection. Cancer Cell.

[62] Hou W., Chen H., Rojas J., Sampath P., Thorne S.H. (2014). Oncolytic vaccinia virus demonstrates antiangiogenic effects mediated by targeting of VEGF. Int. J. Cancer.

[63] Benencia F., Courreges M.C., Conejo-garcía J.R., Buckanovich R.J., Zhang L.I.N., Carroll R.H., Morgan M.A., Coukos G. (2005). Oncolytic HSV Exerts Direct Antiangiogenic Activity in Ovarian Carcinoma. Hum. Gene Ther..

[64] Kurozumi K., Hardcastle J., Thakur R., Shroll J., Otsuki A., Chiocca E.A., Kaur B. (2008). Oncolytic HSV-1 infection of tumors induces angiogenesis and upregulates CYR61. Mol. Ther..

[65] Aghi M., Rabkin S.D., Martuza R.L. (2007). Angiogenic response caused by oncolytic herpes simplex virus-induced reduced thrombospondin expression can be prevented by specific viral mutations or by administering a thrombospondin-derived peptide. Cancer Res..

[66] Rizzo R., D’Accolti M., Bortolotti D., Caccuri F., Caruso A., Di Luca D., Caselli E. (2018). Human Herpesvirus 6A and 6B inhibit in vitro angiogenesis by induction of Human Leukocyte Antigen G. Sci. Rep..

[67] Yan W., Kitzes G., Dormishian F., Hawkins L., Sampson-Johannes A., Watanabe J., Holt J., Lee V., Dubensky T., Fattaey A. (2003). Developing Novel Oncolytic Adenoviruses through Bioselection. J. Virol..

[68] Puig-Saus C., Gros A., Alemany R., Cascalló M. (2012). Adenovirus i-leader truncation bioselected against cancer-associated fibroblasts to overcome tumor stromal barriers. Mol. Ther..

[69] Puig-Saus C., Laborda E., Rodríguez-García A., Cascalló M., Moreno R., Alemany R. (2014). The combination of i-leader truncation and gemcitabine improves oncolytic adenovirus efficacy in an immunocompetent model. Cancer Gene Ther..

[70] Lopez M.V., Viale D.L., Cafferata E.G.A., Bravo A.I., Carbone C., Gould D., Chernajovsky Y., Podhajcer O.L. (2009). Tumor Associated Stromal Cells Play a Critical Role on the Outcome of the Oncolytic Efficacy of Conditionally Replicative Adenoviruses. PLoS ONE.

[71] Viale D.L., Cafferata E.G., Gould D., Rotondaro C., Chemajovsky Y., Curiel D.T., Podhajcer O., Lopez M.V. (2013). Therapeutic improvement of a stroma-targeted CRAd by incorporating motives responsive to the melanoma microenvironment. J. Invest. Dermatol..

[72] José A., Rovira-Rigau M., Luna J., Giménez-Alejandre M., Vaquero E., García De La Torre B., Andreu D., Alemany R., Fillat C. (2014). A genetic fiber modification to achieve matrix-metalloprotease-activated infectivity of oncolytic adenovirus. J. Control. Release.

[73] Freedman J.D., Duffy M.R., Lei-Rossmann J., Muntzer A., Scott E.M., Hagel J., Campo L., Bryant R.J., Verrill C., Lambert A. (2018). An Oncolytic Virus Expressing a T-cell Engager Simultaneously Targets Cancer and Immunosuppressive Stromal Cells. Cancer Res..

[74] De Sostoa J., Fajardo C.A., Moreno R., Ramos M.D., Farrera-Sal M., Alemany R. (2019). Targeting the tumor stroma with an oncolytic adenovirus secreting a fibroblast activation protein-targeted bispecific T-cell engager. J. Immunother. Cancer.

[75] Huang T., Wang H., Chen N.G., Frentzen A., Minev B., Szalay A.A. (2015). Expression of anti-VEGF antibody together with anti-EGFR or anti-FAP enhances tumor regression as a result of vaccinia virotherapy. Mol. Ther. Oncolytics.

[76] Öhlund D., Handly-Santana A., Biffi G., Elyada E., Almeida A.S., Ponz-Sarvise M., Corbo V., Oni T.E., Hearn S.A., Lee E.J. (2017). Distinct populations of inflammatory fibroblasts and myofibroblasts in pancreatic cancer. J. Exp. Med..

[77] Hong C.S., Fellows W., Niranjan A., Alber S., Watkins S., Cohen J.B., Glorioso J.C., Grandi P. (2010). Ectopic matrix metalloproteinase-9 expression in human brain tumor cells enhances oncolytic HSV vector infection. Gene Ther..

[78] Sette P., Amankulor N., Li A., Marzulli M., Leronni D., Zhang M., Goins W.F., Kaur B., Bolyard C., Cripe T.P. (2019). GBM-Targeted oHSV Armed with Matrix Metalloproteinase 9 Enhances Anti-tumor Activity and Animal Survival. Mol. Ther. Oncolytics.

[79] Schäfer S., Weibel S., Donat U., Zhang Q., Aguilar R.J., Chen N.G., Szalay A.A. (2012). Vaccinia virus-mediated intra-tumoral expression of matrix metalloproteinase 9 enhances oncolysis of PC-3 xenograft tumors. BMC Cancer.

[80] Hawinkels L.J.A.C., Zuidwijk K., Verspaget H.W., de Jonge-Muller E.S.M., van Duijn W., Ferreira V., Fontijn R.D., David G., Hommes D.W., Lamers C.B.H.W. (2008). VEGF release by MMP-9 mediated heparan sulphate cleavage induces colorectal cancer angiogenesis. Eur. J. Cancer.

[81] Fingleton B. (2006). Matrix metalloproteinases: Roles in cancer and metastasis. Front. Biosci..

[82] Mahller Y., Vaikunth S., Ripberger M., Baird W., Saeki Y., Cancelas J., Crombleholme T., Cripe T. (2008). Tissue inhibitor of metalloproteinase-3 via oncolytic herpesvirus inhibits tumor growth and vascular progenitors. Cancer Res..

[83] Paolillo M., Schinelli S. (2019). Extracellular matrix alterations in metastatic processes. Int. J. Mol. Sci..

[84] Zhang T., Suryawanshi Y.R., Szymczyna B.R., Essani K. (2017). Neutralization of matrix metalloproteinase-9 potentially enhances oncolytic efficacy of tanapox virus for melanoma therapy. Med. Oncol..

[85] Kreuger J., Spillmann D., Li J.P., Lindahl U. (2006). Interactions between heparan sulfate and proteins: The concept of specificity. J. Cell Biol..

[86] Bejarano M.T., Merchan J.R. (2015). Targeting tumor vasculature through oncolytic virotherapy: Recent advances. Oncolytic Virotherapy.

[87] Jing Y., Tong C., Zhang J., Nakamura T., Iankov I., Russell S.J., Merchan J.R. (2009). Tumor and vascular targeting of a novel oncolytic measles virus retargeted against the urokinase receptor. Cancer Res..

[88] Liu T.C., Zhang T., Fukuhara H., Kuroda T., Todo T., Canron X., Bikfalvi A., Martuza R.L., Kurtz A., Rabkin S.D. (2006). Dominant-negative fibroblast growth factor receptor expression enhances antitumoral potency of oncolytic herpes simplex virus in neural tumors. Clin. Cancer Res..

[89] Saito K., Khan K., Sosnowski B., Li D., O’malley B.W. (2009). Cytotoxicity and antiangiogenesis by fibroblast growth factor 2-targeted Ad-TK cancer gene therapy. Laryngoscope.

[90] Breitbach C.J., Arulanandam R., De Silva N., Thorne S.H., Patt R., Daneshmand M., Moon A., Ilkow C., Burke J., Hwang T.H. (2013). Oncolytic vaccinia virus disrupts tumor-associated vasculature in humans. Cancer Res..

[91] Ulasov I., Borovjagin A.V., Kaverina N., Schroeder B., Shah N., Lin B., Baryshnikov A., Cobbs C. (2015). MT1-MMP silencing by an shRNA-armed glioma-targeted conditionally replicative adenovirus (CRAd) improves its anti-glioma efficacy in vitro and in vivo. Cancer Lett..

[92] Blanco-Prieto S., Barcia-Castro L., Páez de la Cadena M., Rodríguez-Berrocal F.J., Vázquez-Iglesias L., Botana-Rial M.I., Fernández-Villar A., De Chiara L. (2017). Relevance of matrix metalloproteases in non-small cell lung cancer diagnosis. BMC Cancer.

[93] Rao J., Gondi C., Chetty C., Chittivelu S., Joseph P., Lakka S. (2005). Inhibition of invasion, angiogenesis, tumor growth and metastasis by adenovirus-mediated transfer of antisense uPAR and MMP-9 in non-small cell lung cancer cells. Mol. Cancer Ther..

[94] Guse K., Sloniecka M., Diaconu I., Ottolino-Perry K., Tang N., Ng C., Le Boeuf F., Bell J.C., McCart J.A., Ristimäki A. (2010). Antiangiogenic Arming of an Oncolytic Vaccinia Virus Enhances Antitumor Efficacy in Renal Cell Cancer Models. J. Virol..

[95] Duda D.G., Sunamura M., Lozonschi L., Kodama T., Egawa S.I., Matsumoto G., Shimamura H., Shibuya K., Takeda K., Matsuno S. (2000). Direct in vitro evidence and in vivo analysis of the antiangiogenesis effects of interleukin 12. Cancer Res..

[96] Passer B., Cheema T., Wu S., Wu C., Rabkin S., Martuza R. (2013). Combination of vinblastine and oncolytic herpes simplex virus vector expressing IL-12 therapy increases antitumor and antiangiogenic effects in prostate cancer models. Cancer Gene Ther..

[97] Gil M., Seshadri M., Komorowski M.P., Abrams S.I., Kozbor D. (2013). Targeting CXCL12/CXCR4 signaling with oncolytic virotherapy disrupts tumor vasculature and inhibits breast cancer metastases. Proc. Natl. Acad. Sci. USA.

[98] Egen J.G., Ouyang W., Wu L.C. (2020). Human Anti-tumor Immunity: Insights from Immunotherapy Clinical Trials. Immunity.

